# Prosocial Behavior at Work Through the Lens of Character Strengths

**DOI:** 10.3389/fpsyg.2019.03046

**Published:** 2020-01-21

**Authors:** Pavel Freidlin, Hadassah Littman-Ovadia

**Affiliations:** Department of Behavioral Sciences and Psychology, Ariel University, Ariel, Israel

**Keywords:** character strengths, strengths use, prosocial behavior, workplace, organization, workplace outcomes

## Abstract

Character strengths (CSs) are positive traits that have been shown to efficiently and effectively promote a host of positive outcomes, outside and inside the workplace. Despite their theoretical moral basis, they have not been systematically and wholly explored as antecedents of, and correspondingly unused as, mechanisms to increase prosocial behavior (PB) at work. Prosocial behavior at the workplace is desirable, with research pointing to a host of organizational benefits. The utilization of CSs toward PB at work seems like a missed opportunity, given that CSs have been demonstrated as robust positive mechanisms and given that they are characterized by qualities that are accommodating of the complexity of PB: distinct, value-laden, manifest behaviorally, cognitively and emotionally, are plural and sensitive to individual differences and are capable of balancing positive and negative outcomes. The current article will encourage further understanding, examination and implementation of CSs at the workplace, specifically for prosocial purposes, by exploring their conceptual fit and by reviewing initial empirical evidence.

## Introduction

Character strengths (CSs) reflect durable positive individual capacities for feeling, thinking, and behaving, in ways that enable growth and flourishing of individuals and organizations (Peterson and Seligman, 2004). For the past two decades, CS have been widely and robustly researched, demonstrating benefits in a wide variety of fields (Niemiec, 2013; Ruch and Stahlmann, 2019), including the work domain. The good character is seen as allowing optimal functioning and performance in the pursuit of valued and desired outcomes (Peterson and Seligman, 2004; Park and Peterson, 2007). Evidence pointing to CS being efficient and effective mechanisms for promoting positive outcomes at work has been mounting (Miglianico et al., 2019).

Character strength are theorized to have a moral component, as well as to be not diminishing, and elevating of the self and others (Peterson and Seligman, 2004). These assumptions suggest that CS, all or some, should serve as mechanisms capable of explaining and driving prosocial behavior (PB). The work setting is of interest in this respect, as a host of theories, models, reviews and empirical studies emphasize PB’s importance and complexity in this setting (e.g., Bolino and Grant, 2016).

Prosocial behavior is largely beneficial and as such, organizational and vocational scholars are interested in exploring the individual, situational and contextual factors that may explain and subsequently promote it Bolino and Grant (2016). However, insights into its various aspects have been hindered by such conceptual difficulties as defining prosocial motives, or wholly examining prosocial consequences, such as negative ones (Bolino and Grant, 2016).

We suggest adopting an existing, well-researched and comprehensive approach to examine and subsequently promote PB at work. Specifically, we propose implementing the values in action (VIA) classification of CS, introduced by Peterson and Seligman (2004) to describe the good character as an important instance of optimal human functioning at work. Unlike other approaches to strengths [e.g., CliftonStrengths (Rath, 2007) or Strengths Finder (Linley and Bateman, 2018)] the VIA classification focuses on positive character and personality traits in general, such that it isn’t constrained to a specific setting and is widely and robustly researched with published scientific support to its characteristics and benefits (e.g., McGrath, 2017). VIA traits, CS, have been proposed to be prosocial (Peterson and Seligman, 2004; Park and Peterson, 2006) and recent research is providing initial evidence to these claims (e.g., Martínez-Martí et al., 2016). However, CS and PB at work is understudied, as evidenced by it going unmentioned in a recent review of positive outcomes of CS at work (Miglianico et al., 2019). Therefore, we propose to incorporate CS and PB at work. To do so, the following sections will briefly review PB at work and CS at work, and will propose to further capitalize on CS’s benefits by utilizing them to positively promote PB in this setting.

## Prosocial Behavior at Work

“Prosocial Behavior covers the broad range of actions intended to benefit one or more people other than oneself” (Batson, 1998), a common definition in prosociality literature (e.g., Penner et al., 2005; Eisenberg and Spinrad, 2014) used to date (e.g., Baumsteiger, 2019). PB has attracted the attention of a variety of fields but has mostly been assessed through singular and differential approaches (see Penner et al., 2005 for a review), with literature searches revealing next to no studies integrating its emotional, cognitive, and behavioral sources. While differential relationships between different types of antecedents and between types of PB might make sense conceptually, research recognizes that PB cannot be approached from a singular and global perspective and benefits can be reaped by examining its motives and causes in a more nuanced and accommodating manner (Padilla-Walker and Carlo, 2014). This notion highlights that PB is multidimensional, emphasizing the need for a comprehensive approach that encompasses behavioral, cognitive and emotional aspects.

The rarity with which multiple motives to PB are considered was emphasized by Eisenberg and Spinrad (2014), though notions that no sole predictor can account for all PB came earlier. For example, empathy is widely associated with PB, and while it is generally agreed that it is central to PB, empathy doesn’t always lead to PB and research shows that experiencing empathy precedes many but not *all* PB (Penner et al., 2005). Recent studies still examine empathy as a sole predictor of PB (e.g., Smith et al., 2019), without considering additional mechanisms. Other studies focus on cognitive aspects such as intelligence (Guo et al., 2019). Studies that examine multiple predictors do so in a manner that compares the relative contribution of separate mechanisms (e.g., Patrick et al., 2018) or by amalgamating a number of singular, global predictors (e.g., identity and empathy mediation between intelligence and behavior; Guo et al., 2019), rather than providing a wholesome and accommodating approach. Amalgamation introduces issues of decreased explanatory power (Batson, 1998). These singular approaches not only miss the complexity of the mechanisms involved, but are also insensitive to individual differences within PB, as types of PB are different from one another and must be understood as such (Padilla-Walker and Carlo, 2014).

At work, PB is performed by an organization’s member, directed at and in benefit to an individual, group or organization and while one is performing one’s organizational role (Brief and Motowidlo, 1986). In addition to the multidimensionality issue in PB in general, and besides the role constrictions introduced by this more specific definition, Brief and Motowidlo (1986) suggest that PB at work can have both positive *and* negative consequences.

Positive consequences of PB are documented across many studies, such as increases in group performance (Ng and Van Dyne, 2005), in performance appraisals (Podsakoff et al., 2009) and in well-being – of givers, receivers and bystanders (Chancellor et al., 2018).

These benefits not-withstanding, Bolino and Grant (2016) reiterate Brief and Motowidlo (1986) suggestion that prosociality may have darker sides affecting one’s self, others and the organization. For example, interpersonal helping at the workplace was associated with and predictive of emotional exhaustion (Eissa and Lester, 2018). Luthans and Youssef (2007) suggest that *exclusive* focus on positivity can lead to misinterpretations in the organizational setting, propose that positive traits can be *over-exaggerated*, and conclude that positivity must be *balanced*. Given this outlook, and considering both the positive and negative outcomes associated with PB at work, it seems that an approach that would promote PB and its effects in a *balanced* manner, would be beneficial.

Therefore, the examination of PB is in need of an approach that is able to accommodate its cognitive, affective and behavioral aspects, be sensitive to different types of PB and while considering its value-laden nature and potential for positive and negative consequences.

## Character Strengths

Peterson and Seligman (2004) initially identified and defined CS against a number of criteria: (1) Fulfilling, contributing to one’s satisfaction and happiness; (2) Morally valued in its own right, regardless of beneficial outcomes; (3) Not diminishing of others – be elevating and produce admiration; (4) Defined by an obvious negative antonym; (5) Trait-like and manifested in such a way that it can be assessed; (6) Distinctive from other CS; (7) Displayed perfectly in some individuals; (8) Prodigious in that it can be seen in some children and youth; (9) Completely absent in certain individuals; (10) Deliberately cultivated through societal practices and rituals. In addition, traits are culturally and geographically universal and can be developed and learned over one’s lifetime (Peterson and Seligman, 2004).

These criteria were utilized in an extensive, 3-year research project, narrowing down philosophical, scientific and religious literature from the last 2500 years (Peterson and Seligman, 2004). The result is a classification of 24 CS that are categorized across six, universally valued, virtues: (1) Wisdom and knowledge (strengths of creativity, curiosity, judgment, love of learning, and perspective/wisdom); (2) Courage [strengths of bravery, honesty, persistence, and zest (enthusiasm)]; (3) Humanity (strengths of kindness, love, and social intelligence); (4) Justice (strengths of teamwork, fairness, and leadership); (5) Temperance (strengths of forgiveness, modesty, prudence, and self-regulation); and (6) Transcendence (including appreciation of beauty, gratitude, hope, humor, and religiousness). As research continues, virtue categories and CS names are constantly updated based on the new findings on them. For example, the strength of persistence became known as perseverance, and interestingly, has recently been shown to be the most connected CS to performance at the workplace (Littman-Ovadia and Lavy, 2016).

Character strengths are value-laden and are considered the psychological ingredients of “good” character, representing processes or mechanisms by which virtues can be defined (Peterson and Seligman, 2004). Like virtues, CS are thought to intrinsically lead to moral excellence as they are motivational traits that lead to “doing what is right” (Park and Peterson, 2006). As such, they aren’t merely informational as are moral ideals (Peterson and Seligman, 2004), nor are they lacking distinct prosocial content as do some personality traits (Bolino and Grant, 2016).

Initial evidence of CS’s prosocial content exists: the creation of a comprehensive scale of appreciation of beauty and its link to prosociality (Martínez-Martí et al., 2016), examination of the interactions between creativity, moral identity, disengagement and behavioral deviance at work (Zheng et al., 2019), findings that leader moral humility promotes follower moral self-efficacy and their PB (Owens et al., 2019).

Additional insight into CS and prosociality may be had from a recently developed three-factor structure of the VIA classification (see McGrath, 2015 for a review). Namely, the VIA classification divides into components of *inquisitiveness, self-control* and *caring*. The inquisitiveness factor contains strengths such as curiosity and creativity and deals with one’s intellectual endeavors. Factors of self-control and caring are composed of CS that reflect effective functioning in the world (e.g., CS of self-regulation, prudence) and CS reflective of interpersonal and emotional issues (e.g., love and kindness), respectively. All CS are theorized to have a prosocial aspect – morally valued and elevating of the self and *others* (Peterson and Seligman, 2004). However, CS of the *caring* factor are conceptually most directly reflective of PB, and as a full review of all CS and their prosocial aspects is beyond the scope of this review, an overview of the *caring* CS’s (McGrath, 2015) links to PB at work will follow.

For such a review to be round and responsible, it cannot ignore the potential for negative consequences to CS application and prosociality. Recent CS research points to the existence of, and therefore the sensitivity of CS to, positive and *negative* outcomes (e.g., psychopathology, lower well-being; Freidlin et al., 2017; Littman-Ovadia and Freidlin, 2018). CS were proposed to have dark sides (Peterson, 2006) with a practical approach suggesting that CS lie on a continuum, ranging from underusing, through optimally using and finally overusing one’s strengths (Niemiec, 2014). Recent evidence supports this notion through a novel experimental method that assesses CS over and underuse (OUOU; Freidlin et al., 2017; Littman-Ovadia and Freidlin, 2018).

### Forgiveness

Forgiveness reflects the acceptance of others’ shortcomings, letting go of hurt and giving a second chance, not being vengeful (Peterson and Seligman, 2004). In examining effects of leaders’ characters on employees, it was found that forgiving was positively related to how worthy they were of being followed, as well as subordinates’ PB (Liborius, 2014). Underusing this strength may be reflective of *merciless* behavior while its overuse may lead to *permissiveness* – not holding others responsible and allowing everything (Niemiec, 2014; Freidlin et al., 2017).

### Gratitude

Gratitude reflects the experience and expression of thankfulness and not taking things for granted (Peterson and Seligman, 2004). A recent meta-analysis examining gratitude outside the realm of CS pointed to a significant relationship between gratitude and PB (Ma et al., 2017). Gratitude has been examined as the product of PB in the form of reactive helping, with helpers’ receipt of gratitude resulting in higher work engagement and perceived prosocial impact the next day (Lee et al., 2019). These findings could suggest that gratitude at the employee level may be induced by the behavior and perception of higher ranked personnel at work. However, balanced and optimal application would avoid its underuse – *rugged individualism* as well as overuse in the form of *ingratiation* (Niemiec, 2014; Freidlin et al., 2017).

### Humor

Humor is defined by playfulness, making light of difficult situations, making others smile and connecting to others through humor (Peterson and Seligman, 2004). Despite the CS definition, in examining the humor literature itself, it becomes apparent that humor ranges over a wide range of styles, meanings, and motivations (Beermann and Ruch, 2009). Certain styles are deemed as positive: constructive leader humor has been related to more citizenship behaviors – among them helping and PB (Tremblay, 2017). However, humor may have negative consequences (Beermann and Ruch, 2009) that may be represented in humor underuse – *over-seriousness*, or its overuse – *giddiness* (Niemiec, 2014; Freidlin et al., 2017) that could potentially reflect such negative styles as exercising humor at someone’s expense (Beermann and Ruch, 2009).

### Kindness

Kindness incorporates doing good, helping and caring for others, being generous and compassionate (Peterson and Seligman, 2004). Compassion, from an organizational perspective, involves noticing another’s suffering, empathetically feeling another’s pain and acting in a way that will ease the suffering (Lilius et al., 2008). The experience of compassion and caring generates positive outcomes at work, such as greater voluntary helping through the increase of positive emotions and mood (Lilius et al., 2008; Chu, 2016). That being said, attention should be paid to not exhibit the underuse of kindness – *indifference*, or its overuse – *intrusiveness* (Niemiec, 2014; Freidlin et al., 2017).

### Love

Love is characterized by being warm and genuine to, and sharing and accepting love from others, and valuing closeness and intimacy with others (Peterson and Seligman, 2004). This definition suggests that love is limited in its applicability to the workplace and although research is limited, one study examined the encouraging of PB in quality-mentoring relationships, through increased trust and affective commitment, finding increased whistleblowing intentions of antisocial behavior – an organizational PB (Taylor and Curtis, 2013). This suggests that certain aspects of the CS of love, such as trust and intimacy, are applicable and beneficial to the workplace. Attention should be paid to its underuse – *emotional isolation*, while its overuse may lead to *emotional promiscuity*: experiencing no intimacy and experiencing intimacy in inappropriate relationships, respectively (Niemiec, 2014; Freidlin et al., 2017).

### Leadership

Leadership describes a preference to lead rather than follow, to do so by means of positively influencing others, organizing and taking charge to benefit one’s group (Peterson and Seligman, 2004). A meta-analysis comparing various styles of leadership demonstrated that leader influence in general affects employee’s PB, with negligible differences between different leadership styles (Chiaburu et al., 2014). However, caution must be paid to its underuse – *compliancy*, as well as its overuse – *despotism*: not taking charge when one should and taking charge for the sake of power and at cost to others, respectively (Niemiec, 2014; Freidlin et al., 2017).

### Spirituality

Spirituality reflects one having a connection with something larger than oneself, being informed of one’s identity and place in the world through faith, maintaining regular spiritual practices and life being infused with purpose and meaning (Peterson and Seligman, 2004). A study examining the effects of spiritual values among first-line managers found that various components of spirituality are related to PB at work (Ahmed et al., 2019). Attention should be paid to spirituality’s less optimal alternatives: underuse would reflect *anomie* – not believing in a higher power or one’s place in the world, while overuse may lead to *fanaticism* (Niemiec, 2014; Freidlin et al., 2017).

## Discussion and Conclusion

The current paper’s mission is to expand the understanding and encourage the implementation of CS at work, for the purpose of promoting positive PB within it. The state of the current literature largely points to benefits associated with PB at work (Bolino and Grant, 2016), suggesting that the utilization of mechanisms that are capable of promoting such behavior in a balanced manner – is desirable.

Theoretically, the CS framework offers a tight fit to the gaps displayed by the prosocial literature – CS are unified, well-researched, morally-laden, explicit and distinct traits that together make up a flexible, encompassing and at the same time specific classification. Recent developments suggest that CS prescribe optimal levels of behavior, such that their application can account for positive and negative consequences.

Empirical evidence suggests that CS and PB are linked, providing early indications to specific CS being capable of explaining and promoting PB at work. In addition, CS are generally applied through simple and practical interventions (e.g., Kaplan et al., 2014). Given the conceptual fit and empirical evidence, we propose that programs be developed to implement CS at work to not only reap already established work-related benefits (Miglianico et al., 2019), but to promote positive PB. While due to space constraints and the initial nature of the current proposition the current review focused on CS of the caring factor, an examination of all CS and prosociality is warranted. This would be timely as the notion that different types of PB exist is still developing (Padilla-Walker and Carlo, 2014), and future research could capitalize on CS’s plural nature in order to explore both specific and general interactions between CS and PB and gain further understanding of both.

We also suggest a closer examination of CS *use* at work. Specifically, in addition to CS being generally value-laden, the use of CS is mindful of the interaction between one’s dispositions and a given situation. PB seems to be the result of an interaction between situational and dispositional factors as well (Dovidio et al., 2006), and many benefits could be reaped from considering PB through an approach that is sensitive to its positive *and* negative consequences. For example, prosociality targeted at helping a co-worker could have negative consequences for the organization (Bolino and Grant, 2016). From a CS perspective this could reflect the application of a CS, such as kindness, when it is inappropriate to do so, resulting in CS overuse. Further CS research that includes its negative aspects would provide for a more balanced application of CS at work, to further capitalize on existing benefits, prosocially and beyond.

## Author Contributions

All authors listed have made a substantial, direct and intellectual contribution to the work, and approved it for publication.

## Conflict of Interest

The authors declare that the research was conducted in the absence of any commercial or financial relationships that could be construed as a potential conflict of interest.
